# Perceiving Animacy From Deformation and Translation

**DOI:** 10.1177/2041669517707767

**Published:** 2017-05-17

**Authors:** Takahiro Kawabe

**Affiliations:** NTT Communication Science Laboratories, Nippon Telegraph and Telephone Corporation, Japan

**Keywords:** biological motion, body perception, motion, perception, shapes or objects

## Abstract

In a cartoon, we often receive an animacy impression from a dynamic nonanimate object, such as a sponge or a flour sack, which does not have an animal-like shape. We hypothesize that the animacy impression of a nonanimal object could stem from dynamic patterns that are possibly fundamental for biological motion perception. Here we show that observers recognize the animacy of human jump actions from the combination of deformation and translation. We extracted vertical motion vectors from the uppermost and lowermost points in point-light jumper stimuli and assigned the vectors to a uniform rectangle. The participants’ task was to rate the animacy and jump impressions for the rectangle. Results showed that both animacy and jump impressions for the rectangle movements were comparable to those for the original point-light movements. The impressions decreased for stimuli having a deformation or translation component alone, which was extracted from the original motion vectors. By mathematically simulating deformation and translation in a human jump, we also found that the temporal relation between deformation and translation plays a critical role in the determination of jump impressions but only has a moderate effect for animacy impressions. On the basis of the results, we discuss how cartoon techniques take advantage of the properties of biological motion perception.

## Introduction

An animator deftly gives an animacy impression to nonanimate objects in a cartoon. As a classical exercise, beginner animators often try to give such animacy impressions as dejection, joy, curiosity, and laughter to a flour sack (Thomas, 1995).

A critical point of interest is that such nonanimate objects with an animacy impression do not always have an animal-like shape and spatial structure. Namely, an object, such as a flour sack, is seen as alive even when the sack lacks an animal-like body structure. Many previous studies on biological motion perception have argued that the perception of the shape and spatial structure of animals’ appearances plays a key role in perceiving biological motion (Beintema & Lappe, 2002; Lange & Lappe, 2006; Lu, 2010; Thurman & Lu, 2014). It is thus possible that the animacy impression of nonanimate objects in a cartoon stems from sources of visual signals other than an animal-like shape.

Deformation is one promising source of visual signals that contributes to the animacy impression of nonanimate objects in a cartoon. In the flour sack exercise described earlier, the sack is given various kinds of dynamic deformation such as squashing, stretching, and twisting (Thomas, 1995). Although some previous studies have reported the role of image motion signals in biological motion perception (Blake & Shiffrar, 2007; Burr, Ross, & Morrone, 1986, Chang & Troje, 2008; Giese & Poggio, 2003, Troje, 2002, Troje & Westhoff, 2006), it has not been well documented which visual information in dynamic deformation determines the perception of animacy and biological activities.

Previous studies have suggested that dynamic deformation can elicit an animacy impression. There is a long history of research that demonstrates animacy from nonanimate objects whose static appearances are far from animal (Scholl & Tremoulet, 2000 give an excellent review). Heider and Simmel (1944) created a display in which several geometrical figures (a large triangle, a small triangle, and a small circle) move around a large rectangle. A representative dynamic pattern of the figures’ movements is as if the large triangle chases the small triangle and the small circle. Viewing such a display, the observers tend to have animacy impressions for the figures. Michotte (1963) reported that when a square elongates and contracts rhythmically while translating in space, an observer sees an animal-like impression for the square. The stimulus is called a Caterpillar stimulus. By using Caterpillar stimuli, Schlottmann and Surian (1999) showed that 9-month-old infants habituated to a visual event wherein the Caterpillar stimulus moved toward a target. When a temporal relation between the Caterpillar stimulus and the onset of a target motion was manipulated, infants habituated selectively to the conditions wherein the target started moving before and after the Caterpillar stimulus stopped, respectively. Schlottmann and Surian indicated that this evidence suggested that infants are sensitive to a causation-at-a-distance. Later studies have shown that 6-month-old infants as well as children aged 3 to 7 years old also recognized the causation-at-a-distance on the basis of a relation between the Caterpillar stimulus and its target (Schlottmann, Cole, Watts, & White, 2013; Schlottmann, & Ray, 2010). The Caterpillar stimulus contains both deformation and translation components. On the other hand, it has remained unclear what visual information in stimuli like the Caterpillar stimulus could be used as a cue to an animacy impression.

The present study examined how visual information in the movement of nonanimate objects could contribute to the animacy and jump impressions. Among a variety of biological motions to choose from, we focused on a human jump action as the target because a human jump action is considered one of the common repertoires of human actions. In addition, a human jump action consists of a combination of deformation and translation that are mathematically separable from each other (Blickhan, 1989). As such, jump actions are likely useful to clarify the visual information that is necessary for an animacy impression. From traditional point-light stimuli (Johansson, 1973), we extracted the one-dimensional vertical motion vectors of the uppermost and lowermost point lights and assigned the motion vectors to the top and bottom side of a rectangle, respectively. We focused on not only jump impressions but also animacy impressions because a previous study has shown a tight relationship between animacy impressions and biological motion perceptibility (Chang & Troje, 2008; Schultz, & Bülthoff, 2013). As a result, we found that the deforming rectangle could produce both animacy and jump impressions that were statistically comparable to the traditional point-light stimuli. Moreover, in accordance with the previous approach, in which biological motion signals were decomposed into translation and articulated motion (Masselink & Lappe, 2015), we decomposed the movement pattern of the rectangle into elementary components (pure deformation and pure translation) and tried to identify whether each of the components alone could also produce a strong impression of animacy and jump.

In Experiments 1 and 2, we tested whether the animacy and jump impressions of the point-light jumper could be explained by its deformation and translation components and their combination. In Experiment 3, we examined how the relative timing between deformation and translation affects the animacy and jump impressions.

## Experiment 1

### Method

#### Observers

Ten naïve observers participated in this experiment. All observers in this study were unaware of the specific purpose of the experiments. They reported having normal or corrected-to-normal visual acuity. Participants were paid for their participation. Ethical approval for this study was obtained from the ethical committee at Nippon Telegraph and Telephone Corporation (NTT Communication Science Laboratories Ethical Committee). The experiments were conducted according to the principles laid down in the Helsinki Declaration. Written informed consent was obtained from all participants.

#### Apparatus

Stimuli were presented on a 21-in. CRT monitor (GDM-F500R, Sony) with a resolution of 1024 × 768 pixels and a refresh rate of 60 Hz. The luminance emitted from the monitor was linearized in a range from 0 to 132 cd/m^2^ using a photometer (OP200-E, Cambridge Research Systems). A computer (Windows 7 32-bit OS) controlled stimulus presentation and data collection using PsychoPy v1.83 (Peirce, 2007, 2009).

#### Stimuli

**Figure 1.**
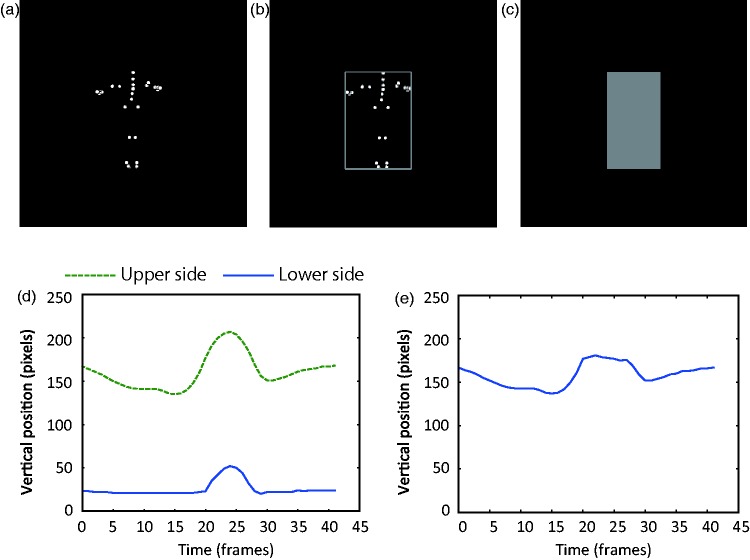
Stimuli used in Experiment 1 and the analysis of motion patterns in the stimuli. (a) A snapshot of a point-light jumper. (b) A point-light jumper with a bounding box. (c) Filled bounding box of (b). (d) Vertical position shift of upper and lower sides of the bounding box. (e) Deformation component as calculated by subtracting the motion vector of the lower side of the box jumper from the motion vector of the upper side. Here, the initial vertical position of the upper side is added to the differential components.

Conventional point-light *jumper* stimuli were created, and from the stimuli, we tried to extract one-dimensional vertical motion patterns that were related to deformation and translation of an overall jumper figure. To create the point-light jumper stimuli, Carnegie-Mellon Graphics Lab Motion Capture Database (http://mocap.cs.cmu.edu/) was utilized. As shown in Figure 1(a), the camera position was set to capture the whole body of each jumper and locate the initial body center of the jumper at the center of an image region in stimuli (Supplementary Video 1). In total, 6-point-light stimuli were captured, each of which was derived from each of the six jumper IDs in the database: 02_04, 13_39, 16_1, 49_02, 118_01, and 131_07. Mean values (standard deviation) for the width and height of the six jumpers in the stimulus videos were 74.26 (10.92) and 142.08 (13.80) pixels, respectively. The point-light jumper videos lasted for 1.507 s on average.

To extract the one-dimensional vertical motion patterns, a bounding box was added to a point-light jumper on each frame (Figure 1(b)), and the magnitude of the vertical position shift for the upper and lower side of the bounding box (Figure 1(d)) was calculated. The extracted motion vectors were assigned to the upper and lower sides of the rectangle with a neutral gray (66 cd/m^2^) surface. The rectangle stimuli are referred to as *box jumpers* (Figure 1(c), Supplementary Video 2). In this way, a set of box jumper stimuli was obtained that had the motion vectors of the uppermost and lowermost points of the point-light jumper. The width of the box jumpers was kept constant at 2.2° of visual angle because the point of interest was the vertical position shift of the upper and lower side of the bounding box. The mean height of the jumpers was 4.26° (with 0.41° of standard deviation). The jumpers and boxes were presented against a black background (0 cd/m^2^). The stimulus movie for each condition lasted 1.51 s on average with a standard deviation of 0.56 s.

Also of interest is whether deformation or translation components for the motion patterns of the box jumper were sufficient to trigger the animacy and jump impressions comparably to the point-light jumpers and box jumpers. As shown in Figure 1(d), from the beginning of a video, the upper side of the box jumper moved in the vertical direction, while the lower side of the rectangle did not move until the 20th frame. After 28 frames, again the upper side started to shift, while the lower side did not. The assumption was that the vertical position shifts of the upper side of the box jumper occurred due to the deflection of the body, which was caused by an applied force, and could thus be considered as deformation. The deformation components were obtained by subtracting the vertical position of the lower side from the upper side (see Figure 1(e) and Supplementary Video 3, wherein the initial vertical position of the upper side is added to the calculation outcome). On the other hand, both the upper and lower sides caused a vertical position shift between the 20th and 28th frames. The assumption was that the shared position shift came from parabolic translation during an aerial phase in a jump. The vertical position shift of the lower side was considered to be the translation component that was shared with the upper side, because the shift came from pure parabolic translation, and it was expected that both the upper and lower sides would move by an identical amount (see the Method section of Experiment 2 for the rationale for this line of thought). Thus, the vertical position shift of the lower side of the box jumper was considered to be the *translation* component. A *reversed* condition was also tested (Supplementary Video 4 and Video 5), in which the motion vectors of the deformation components were reversed. The visual system was tested under this condition to see whether it could utilize the proper combination of deformation and translation.

### Procedure

The experiments were conducted in a dimly lit room. Each observer was tested individually and was asked to view the stimuli at 70 cm from the CRT monitor. Pressing the spacebar on the keyboard of the computer started each trial. After 500 ms, a stimulus video was presented. After the video had disappeared, observers were asked to rate the animacy and jump impressions on a 5-point scale, where “5” means a strong impression and “1” means no impression, with 4, 3, and 2 indicating an impression somewhere between strong and none. They reported their scores by pressing corresponding digit keys on the keyboard. Each point-light jumper and its box jumper were repeatedly assessed eight times. Thus, each observer received two sessions of 240 trials consisting of 5 jumper conditions (point-light jumpers, box jumpers, deformation, translation, and reversed) × 6 jumpers × 8 repetitions. The impressions of animacy and jump were assessed in separate sessions. The order of the two sessions was randomized across the observers. It took approximately 20 min to complete each session.

### Results and Discussion

**Figure 2.**
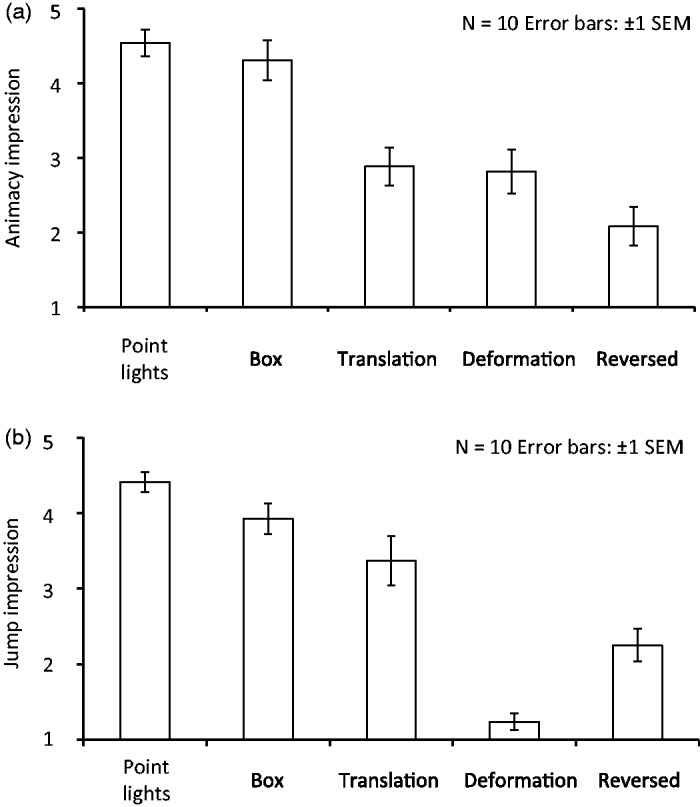
Experimental results. Ratings of (a) animacy and (b) jump impressions are plotted for each condition.

Calculated mean rating scores of the animacy and jump impressions are shown in Figure 2(a) and (b). To confirm the statistical significance of differences among all conditions, a one-way repeated measures analysis of variance (ANOVA) was separately conducted for the scores of the animacy and jump impressions. Figure 2(a) shows the mean scores of the animacy impression for each of the conditions. The main effect of a one-way repeated ANOVA was significant, *F*(4, 36) = 25.64, *p* < .0001, partial η^2 ^= 0.74. The box jumpers were expected to have lower impressions of animacy than the point-light jumpers because a large part of the detailed structure in the original point-light jumpers was lost in the box jumpers. Surprisingly, however, multiple comparison tests showed that the animacy impression was comparable between the point-light and box jumpers (*p* > .1). Another goal was to determine whether decomposed deformation and translation were sufficient cues to cause the impressions. The scores in the deformation and translation conditions were significantly different from those in the point-light jumper and box jumper conditions (*p* < .0001). The impressions between deformation and translation were not statistically different (*p* > .1). A final condition tested whether the inconsistency between deformation and translation had a detrimental effect on the animacy impressions (see Method section for the rationale for using this condition). When motion vectors were inconsistent between deformation and translation components, the score was significantly different from that for the point-light jumper and box jumper conditions (*p* < .0001).

The results showed that the observers perceived strong animacy impressions for the box jumpers with one-dimensional vertical motion patterns extracted from the point-light jumpers. Surprisingly, the strength of impressions was comparable between the point-light and box jumpers. Moreover, neither the deformation nor translation produced animacy impressions comparable to the box jumpers. The results indicate that human observers utilize the combination of deformation and translation to recognize animacy in a human-jump scene.

Figure 2(b) shows the mean scores of the jump impressions for each condition. The scores for the jump impression were analyzed in a similar way as for the animacy impression. In the one-way repeated measures ANOVA, the main effect was significant, *F*(4, 36) = 52.068, *p* < .0001, partial η^2 ^= 0852. Multiple comparison tests showed that the impression results between the point-light and box jumpers were again not significantly different (*p* > .1). Moreover, the impression results between the box jumper and translation were not statistically different (*p* > .1). For other pairs, the scores were significantly different (at least *p* < .0001).

The results indicate that the observers reported a strong jump impression for box jumpers. On the other hand, the translation condition also produced a relatively strong impression of jumping. The results indicate that the parabolic translation might be a sufficient cue for observers to judge whether a human agent is jumping.

The results for the reversed condition may indicate the necessity of the appropriate combination between deformation and translation in forming animacy and jump impressions. The rating scores for this condition were significantly lower than the scores for the box jumper condition. Interestingly, the decrease in the scores was observed for both animacy and jump impressions. The results indicate that human observers evaluate the consistency of motion vectors between deformation and translation to determine the animacy and jump impression. Hence, this suggests, at least, that the observers exploited the consistency of deformation and translation to judge whether the movement was related to an animated entity or not and whether it came from a human jump.

An important issue to address was whether the viewing experiences had possibly confounded the results. The observers viewed the point-light jumper stimuli as well as other stimuli during the same session. Because the point-light jumper stimuli had partly identical spatiotemporal profiles to the box jumper stimuli, it was possible that the observers might have been ready to imagine the point-light jumper when viewing the box-jumper. Due to the viewing experiences, the box jumpers might have been given higher rating scores of animacy and jump impressions than other stimuli conditions. To rule out the possibility, the next experiment was conducted in which the point-light jumper stimuli were eliminated from a stimulus list.

## Experiment 2

### Method

#### Observers

Ten naïve observers, who did not participate in Experiment 1, participated in this experiment.

#### Apparatus

The apparatus was identical to that used in Experiment 1.

#### Stimuli

In this experiment, the point-light jumper condition as used in Experiment 1 was omitted. The other four conditions were tested: the box, translation, deformation, and reversed conditions. The properties of the stimuli were exactly identical to those used in Experiment 1.

#### Procedure

The procedure was also identical to that used in Experiment 1 except for the following. Each observer received two sessions of 192 trials consisting of 4 jumper conditions (box jumpers, deformation, translation, and reversed conditions) × 6 jumpers × 8 repetitions. The impressions of animacy and jump were assessed in separate sessions. The order of the two sessions was randomized across the observers. It took approximately 20 min to complete each session.

### Results and Discussion

**Figure 3.**
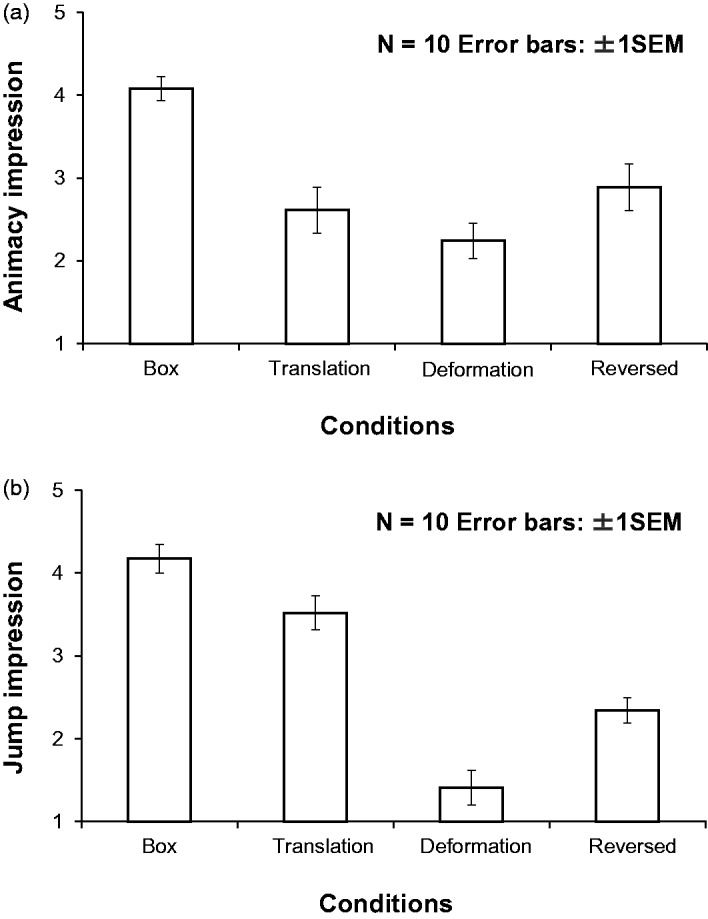
The results of Experiment 2. Ratings of (a) animacy and (b) jump impressions are plotted for each condition.

Calculated mean rating scores of animacy and jump impressions are shown in Figure 3(a) and (b). To confirm the statistical significance of differences among all conditions, a one-way repeated measures ANOVA was again conducted separately for the scores of the animacy and jump impressions. Figure 3(a) shows the mean scores of the animacy impression for each of the conditions. The main effect of the stimulus conditions was significant, *F*(3, 27) = 10.29, *p* < .0001, partial η^2 ^= 0.533. Multiple comparison tests showed that the rating scores in the box jumper condition were significantly higher than the other three conditions (at least *p* < .005). Figure 3(b) shows the mean scores of jump impressions for each condition. In the one-way repeated measures ANOVA, the main effect was significant, *F*(3, 27) = 52.762, p < .0001, partial η^2 ^= 0854. Multiple comparison tests showed that each condition was significantly different from other conditions (*p* < .05).

The results indicate that the box jumper stimuli still caused stronger impressions of animacy and jump than the other stimulus conditions, even when the experience of viewing the point-light jumper stimuli was excluded. Thus, this suggests that the advantage of the box jumper condition over other conditions in determining animacy and jump impressions does not stem from the experience of viewing the point-light jumper stimuli.

Experiments 1 and 2 indicated that the appropriate combination of deformation and translation contributed to an animacy impression of human jump actions. The next experiment explores how relative timing between deformation and translation could modulate the animacy and jump impressions.

## Experiment 3

### Method

#### Observers

The 10 naïve observers who had participated in Experiment 1 also participated in this experiment including a jump impression rating task. In addition, 8 of the 10 observers participated in this experiment including an animacy impression rating task. They were still unaware of the specific purpose of the experiment.

#### Apparatus

The apparatus was identical to that used in Experiment 1.

#### Stimuli

**Figure 4.**
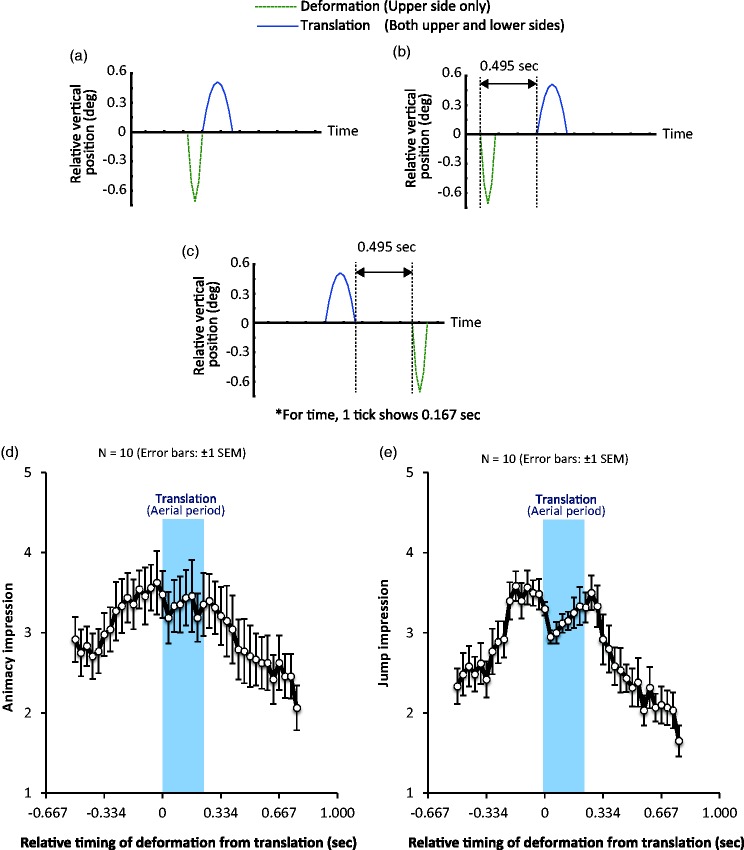
Relative vertical position shifts for deformation (green dotted lines) and the translation (blue solid lines) components in (a) a natural setting condition, (b) a deformation condition that precedes the onset of the translation by 0.495 s, and (c) a deformation condition that follows 0.495 s after the offset of translation. (d) Ratings of the jump impression as a function of relative temporal onsets of deformation relative to translation.

Using only the box jumper conditions, the temporal relation between the deformation and translation components was manipulated. The spring-mass model proposed by Blickhan (1989) was employed to simulate a human jump, and the vertical position shift due to deformation and translation was independently calculated. In a spring-mass system with a linear spring, a jump has two consecutive phases: a contact phase and an aerial phase. During the contact phase, a force is applied to a body, and a deflection occurs whose magnitude reaches the maximum degree at the temporal middle point of the contact phase. After the contact phase, the aerial phase starts, in which a body translates in a parabolic manner. Based on the description of the jump movement in a spring-mass system, it is assumed that the image information about a jump movement can be decomposed into the following two features: A deformation caused by the deflection of a body and a translation caused by a parabolic aerial movement. The vertical deflection *y* in the contact phase was calculated by using the following formula:

(1) $$y=\frac{\overset{\cdot }{y}a}{\omega }\sin\omega t-\frac{g}{\omega^{2}}\cos\omega t+\frac{g}{\omega^{2}}$$

where $\overset{\cdot }{y}a$ is vertical velocity and *t* is time. In Formula (1),

(2) $$\omega^{2}=\frac{k}{m}$$

where *k* is stiffness (30 × 10^3^) and *m* is mass (65 kg). The translation in the aerial phase was calculated by using the following simple formula:

(3) $$y=\upsilon_{0}t-\frac{1}{2}{\mathrm{gt}}^{2}$$

where $\upsilon_{0}$ is initial vertical velocity, and *g* is gravitational acceleration. From the calculations, we obtained the vertical position shift due to deformation, which lasted 0.132 s, and the vertical position shift due to translation, which lasted 0.264 s (Figure 4). As shown in Figure 4(a), in a natural setting, the image deformation starts 0.132 s earlier than the onset of translation. Here, it is assumed that the vertical shifts of deformation occur at the upper side of the box jumpers, while the vertical shifts of translation occur at both the upper and lower sides. The relative temporal onset of the deformation relative to the translation was manipulated. In one side of the extreme condition (Figure 4(b)), the deformation started 0.495 s earlier than the onset of the translation. On the other side of the extreme condition (Figure 4(c)), the image deformation started 0.495 s after the temporal offset of translation. The period between these extreme relative onset conditions was subdivided into 39 levels. The width of the box jumper was kept constant at 2.2°. The initial height of the jumpers was kept constant at 4.26°.

### Procedure

The procedure was identical to that used in Experiment 1, except that each condition was repeated six times, and each observer therefore performed 234 trials consisting of 39 relative onset conditions × 6 repetitions. The observers again rated their animacy and jump impressions.

### Results and Discussion

Calculated mean rating scores of animacy and jump impressions for each temporal onset are plotted in Figure 4(d) and (e), respectively. Using the scores, a one-way repeated measures ANOVA was conducted with the relative onset condition as the within-subject factor. For the animacy impressions, the main effect was significant, *F*(38, 342) = 5.695, *p* < .0001, partial η^2 ^= 0.448. For the jump impression, the main effect was also significant, *F*(38, 342) = 11.984, *p* < .0001, partial η^2 ^= 0.57.

For the animacy impression, the scores had a dull peak when the relative timing for the onset of deformation from the one of translation was −0.33 s. The results suggest that the animacy impression is moderately attuned to the relative timing between translation and onsets. On the other hand, the maximum score itself (3.625 on average) was not as high as the scores observed in the box jumper conditions of Experiment 1 (3.927) and Experiment 2 (4.08). The results indicate that the approximation of human jump action by using a spring-mass model may ignore some aspects of visual information that possibly enhance an animacy impression.

For the jump impression, on the other hand, the scores had two peaks. The first peak appeared at relative onsets ranging from −0.2 to 0 s, which is consistent with the idea that human observers are sensitive to the relative temporal timing between deformation and translation for seeing a jump. The other peak was also observed when image deformation occurred at relative onsets ranging from 0.165 to 0.297 s, which were near the temporal offset of translation. As shown in Figure 1(d), deflection due to an applied force occurs at both the temporal onset and offset of translation in the aerial period of a human jump. Thus, the visual system possibly takes advantage of knowledge about how a human body shape changes when the feet contact the ground after a jump, and it utilizes the knowledge to judge whether the movement in a scene comes from a human jump.

## General Discussion

The present study showed that the box jumper elicited animacy and jump impressions comparable to those for the point-light jumper. The results indicate that human observers extract the combination of deformation and translation from the complex motion pattern of biological motion and exploit it to judge animacy and jumping. Moreover, we observed that the temporal consistency between deformation and translation was interpreted as a sign of human jumping. Thus, the temporal grouping between deformation and translation is possibly utilized by the visual system to judge jump impressions. On the other hand, the temporal consistency influenced animacy impressions only moderately. The results indicate that the perception of animacy from the combination of deformation and translation is possibly mediated by a mechanism that is different from, or partly shared by, the mechanism for the perception of the human jump itself.

The results also indicate that the visual system utilizes knowledge about biophysical changes in body structures to judge animacy and jump impressions. It is known that the human visual system utilizes knowledge about the anatomical possibility of human body movements in interpreting the direction of a two-frame apparent motion (Shiffrar & Freyd, 1990, 1993). That is, human observers tend to perceive motion paths that are plausible in terms of the anatomical movability of the arm around a joint. In a similar way, the results suggest that the visual system possesses some naïve knowledge about the possible deformation and translation of the human body, which can be described by a spring-mass model as proposed by Blickhan (1989; see also Dickinson et al. [2000] for an excellent review for biophysical descriptions of animal motion). The system might use the naïve knowledge when it tries to interpret what deformation and translation indicate in a scene.

The box jumper only used the combination of deformation and translation. Nevertheless, it was seen as a jumper with animacy, not as a nonanimate soft material such as a rubber box that bounces against a floor. Computationally, image motion is generally categorized into rigid and nonrigid motion, and nonrigid motion is further subdivided into articulated, elastic, and fluid motion (Aggarwal, Cai, Liao, & Sabata, 1994; Huang, 1990). Both the box jumper and a rubber box have rigid motion (which may correspond to parabolic translation) and nonrigid elastic motion (which may correspond to deformation). Thus, both the box jumper and rubber box are categorized into the same class (Aggarwal et al., 1994; Huang, 1990). An important difference between the two is the presence or absence of the self-propulsion of their motion. The role of self-propulsion and self-generation of movements in animacy impressions has been well documented (Cicchino, Aslin, & Rakison, 2011; Dasser, Ulbaek, & Premack, 1989; Scholl & Tremoulet, 2000; Tremoulet & Feldman, 2000). On the other hand, it is possible that self-propulsive motion is not a sufficient factor determining the animacy impression because we observed the low-animacy impressions in some conditions of our experiments (deformation, translation, and reversed conditions of Experiments 1 and 2). Thus, upon a given self-propulsive motion, the visual system might further analyze the pattern in deformation and translation and determine whether an object undergoing deformation and translation is alive or not.

On the basis of the results of the present study, we propose that an animacy impression of nonanimate objects in a cartoon comes from biological motion perception based on the deformation and translation of objects. Here, the patterns of deformation and translation were simplified for experimental purposes. On the other hand, previous animation literature suggests that more complex patterns of deformation such as squashing, stretching, and twisting could cause an animacy and emotional impression for a nonanimate thing such as a flour sack (Thomas, 1995). By psychologically analyzing the effect of complex deformation patterns on the impressions and combining other biological features such as hands, eyes, and mouths, which can likely enhance animacy impressions, we might be able to establish a technique for editing the animacy impression of nonanimate objects without detailed computer graphics or physics simulations. One of the critical limitations of our study is that biological motion stimuli as used here were limited to human jump actions. It is necessary to check in future studies whether the present results can be extended to the biological motion of jump actions of other species.

## Supplementary Material

Supplementary material

Supplementary material

Supplementary material

Supplementary material

Supplementary material

## Supplemental Material

The online supplementary videos are available at http://journals.sagepub.com/doi/suppl/10.1177/2041669517707767.

## References

[bibr1-2041669517707767] Aggarwal, J. K., Cai, Q., Liao, W., & Sabata, B. (1994). Articulated and elastic non-rigid motion: A review. *Proceedings of the 1994 IEEE Workshop on Motion of Non-Rigid and Articulated Objects*, 2–14. Retrieved from http://doi.org/10.1109/MNRAO.1994.346261.

[bibr2-2041669517707767] Beintema, J. A., & Lappe, M. (2002). Perception of biological motion without local image motion. *Proceedings of the National Academy of Sciences, 99*, 5661–5663. Retrieved from http://doi.org/10.1073/pnas.082483699. 10.1073/pnas.082483699 PMC122827 11960019

[bibr3-2041669517707767] Blake, R., & Shiffrar, M. (2007). Perception of human motion. *Annual Review of Psychology, 58*, 47–73. Retrieved from http://doi.org/10.1146/annurev.psych.57.102904.190152. 10.1146/annurev.psych.57.102904.190152 16903802

[bibr4-2041669517707767] Blickhan R. (1989) The spring-mass model for running and hopping. Journal of Biomechanics 22: 1217–1227. 2625422 10.1016/0021-9290(89)90224-8

[bibr5-2041669517707767] Burr D. C. Ross J. Morrone M. C. (1986) Seeing objects in motion. Proceedings of the Royal Society of London. Series B, Containing Papers of a Biological Character 227: 249–265. 10.1098/rspb.1986.0022 2871557

[bibr6-2041669517707767] Chang D. H. F. Troje N. F. (2008) Perception of animacy and direction from local biological motion signals. Journal of Vision 8: 3–3. Retrieved from http://doi.org/10.1167/8.5.3. 10.1167/8.5.3 18842074

[bibr7-2041669517707767] Cicchino J. B. Aslin R. N. Rakison D. H. (2011) Correspondences between what infants see and know about causal and self-propelled motion. Cognition 118: 171–192. Retrieved from http://doi.org/10.1016/j.cognition.2010.11.005. 21122832 10.1016/j.cognition.2010.11.005 PMC3038602

[bibr8-2041669517707767] Dasser V. Ulbaek I. Premack D. (1989) The perception of intention. Science 243: 365–367. 2911746 10.1126/science.2911746

[bibr9-2041669517707767] Dickinson M. H. Farley C. T. Full R. J. Koehl M. A. R. Kram R. Lehman S. (2000) How animals move: An integrative view. Science 288: 100–106. Retrieved from http://doi.org/10.1126/science.288.5463.100. 10753108 10.1126/science.288.5463.100

[bibr10-2041669517707767] Giese M. A. Poggio T. (2003) Neural mechanisms for the recognition of biological movements. Nature Reviews Neuroscience 4: 179–192. Retrieved from http://doi.org/10.1038/nrn1057. 12612631 10.1038/nrn1057

[bibr111-2041669517707767] Heider, F., & Simmel, M. (1944). An Experimental Study of Apparent Behavior. *The American Journal of Psychology*, *57*, 243–259. Retrieved from https://doi.org/10.2307/1416950.

[bibr11-2041669517707767] Huang, T. S. (1990). Modeling, analysis, and visualization of nonrigid object motion. *Proceedings of 10th International Conference on Pattern Recognition*, pp. 361–364, Vol. 1. Retrieved from http://doi.org/10.1109/ICPR.1990.118129.

[bibr12-2041669517707767] Johansson G. (1973) Visual perception of biological motion and a model for its analysis. Perception & Psychophysics 14: 201–211. Retrieved from http://doi.org/10.3758/BF03212378.

[bibr13-2041669517707767] Lange J. Lappe M. (2006) A model of biological motion perception from configural form cues. The Journal of Neuroscience 26: 2894–2906. Retrieved from http://doi.org/10.1523/JNEUROSCI.4915-05.2006. 16540566 10.1523/JNEUROSCI.4915-05.2006 PMC6673973

[bibr14-2041669517707767] Lu, H. (2010). Structural processing in biological motion perception. *Journal of Vision, 10*. Retrieved from http://doi.org/10.1167/10.12.13. 10.1167/10.12.13 21047745

[bibr15-2041669517707767] Masselink J. Lappe M. (2015) Translation and articulation in biological motion perception. Journal of Vision 15: 10, Retrieved from http://doi.org/10.1167/15.11.10. 10.1167/15.11.10 26270192

[bibr16-2041669517707767] Michotte, A. (1963). *The perception of causality* (Vol. xxii). Oxford, England: Basic Books.

[bibr17-2041669517707767] Peirce J. W. (2007) PsychoPy—Psychophysics software in Python. Journal of Neuroscience Methods 162: 8–13. Retrieved from http://doi.org/10.1016/j.jneumeth.2006.11.017. 17254636 10.1016/j.jneumeth.2006.11.017 PMC2018741

[bibr18-2041669517707767] Peirce J. W. (2009) Generating stimuli for neuroscience using PsychoPy. Frontiers in Neuroinformatics 2: 10, Retrieved from http://doi.org/10.3389/neuro.11.010.2008. 19198666 10.3389/neuro.11.010.2008 PMC2636899

[bibr19-2041669517707767] Schlottmann, A., Cole, K., Watts, R., & White, M. (2013). Domain-specific perceptual causality in children depends on the spatio-temporal configuration, not motion onset. *Frontiers in Psychology, 4*. Retrieved from https://doi.org/10.3389/fpsyg.2013.00365. 10.3389/fpsyg.2013.00365 PMC3708160 23874308

[bibr20-2041669517707767] Schlottmann A. Ray E. (2010) Goal attribution to schematic animals: Do 6-month-olds perceive biological motion as animate? Developmental Science 13: 1–10. Retrieved from https://doi.org/10.1111/j.1467-7687.2009.00854.x. 20121858 10.1111/j.1467-7687.2009.00854.x

[bibr21-2041669517707767] Schlottmann A. Surian L. (1999) Do 9-month-olds perceive causation-at-a-distance? Perception 28: 1105–1113. Retrieved from https://doi.org/10.1068/p281105. 10694960 10.1068/p281105

[bibr22-2041669517707767] Scholl B. J. Tremoulet P. D. (2000) Perceptual causality and animacy. Trends in Cognitive Sciences 4: 299–309. Retrieved from https://doi.org/10.1016/S1364-6613(00)01506-0. 10904254 10.1016/s1364-6613(00)01506-0

[bibr23-2041669517707767] Schultz, J., & Bülthoff, H. H. (2013). Parametric animacy percept evoked by a single moving dot mimicking natural stimuli. *Journal of Vision, 13*. Retrieved from http://doi.org/10.1167/13.4.15. 10.1167/13.4.15 23525131

[bibr24-2041669517707767] Shiffrar M. Freyd J. J. (1990) Apparent motion of the human body. Psychological Science 1: 257–264. Retrieved from http://doi.org/10.1111/j.1467-9280.1990.tb00210.x.

[bibr25-2041669517707767] Shiffrar M. Freyd J. J. (1993) Timing and apparent motion path choice with human body photographs. Psychological Science 4: 379–384. Retrieved from http://doi.org/10.1111/j.1467-9280.1993.tb00585.x.

[bibr26-2041669517707767] Thomas F. (1995) The illusion of life, New York, NY: Disney Book Group.

[bibr27-2041669517707767] Thurman S. M. Lu H. (2014) Bayesian integration of position and orientation cues in perception of biological and non-biological forms. Frontiers in Human Neuroscience 8: 91, Retrieved from http://doi.org/10.3389/fnhum.2014.00091. 24605096 10.3389/fnhum.2014.00091 PMC3932410

[bibr28-2041669517707767] Tremoulet P. D. Feldman J. (2000) Perception of animacy from the motion of a single object. Perception 29: 943–951. 11145086 10.1068/p3101

[bibr29-2041669517707767] Troje N. F. (2002) Decomposing biological motion: A framework for analysis and synthesis of human gait patterns. Journal of Vision 2: 2–2. Retrieved from http://doi.org/10.1167/2.5.2. 10.1167/2.5.2 12678652

[bibr30-2041669517707767] Troje N. F. Westhoff C. (2006) The inversion effect in biological motion perception: Evidence for a “Life Detector”? Current Biology 16: 821–824. Retrieved from http://doi.org/10.1016/j.cub.2006.03.022. 16631591 10.1016/j.cub.2006.03.022

